# Sustainable Development in Rural Underserved Communities through Improved Responsible Management of Decentralized Wastewater Infrastructure: A Focus on the Alabama Black Belt

**DOI:** 10.1021/acs.est.4c01170

**Published:** 2024-10-10

**Authors:** Amal Bakchan, Kevin D. White

**Affiliations:** † Department of Construction Science, Texas A&M University, 3137 TAMU, College Station, Texas 77843, United States; ‡ Department of Civil, Coastal and Environmental Engineering, University of South Alabama, 150 Student Services Drive, SHEC 3142, Mobile, Alabama 36688, United States

**Keywords:** Wastewater, decentralized infrastructure, responsible management entity, operations and maintenance, socio-technical barriers, underserved communities, rural areas, statistical analysis

## Abstract

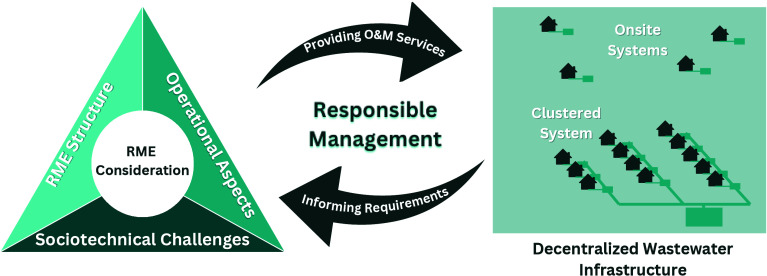

Despite global efforts on meeting sustainable development goals by 2030, persistent and widespread sanitation deficits in rural, underserved communities in high-income countries—including the United States (US)—challenge achieving this target. The recent US federal infrastructure funding, coupled with research efforts to explore innovative, alternative decentralized wastewater systems, are unprecedented opportunities for addressing basic sanitation gaps in these communities. Yet, understanding how to best manage these systems for sustainable operations and maintenance (O&M) is still a national need. Here, we develop an integrated management approach for achieving such sustainable systems, taking into account the utility structure, operational aspects, and possible barriers impeding effective management of decentralized wastewater infrastructure. We demonstrate this approach through a binomial logistic regression of survey responses from 114 public and private management entities (e.g., water and sewer utilities) operating in 27 states in the US, targeting the rural Alabama Black Belt wastewater issues. Our assessment introduces policy areas that support sustainable decentralized wastewater systems management and operations, including privatizing water-wastewater infrastructure systems, incentivizing/mandating the consolidation of utility management of these systems, federally funding the O&M, and developing and retaining water-wastewater workforce in rural, underserved communities. Our discussions give rise to a holistic empirical understanding of effective management of decentralized wastewater infrastructure for rural, underserved communities in the US, thereby contributing to global conversations on sustainable development.

## Abstract 2

The responsible management entity of decentralized wastewater infrastructure systems in small, rural communities is conceptualized based on a local bottom-up approach grounded in what aligns best with communities’ capacity and needs, while accounting for entity structure and operations.

## 1 Introduction

Sustainable Development Goals (SDGs) are the world’s shared agenda for global development that seeks to alleviate poverty by 2030.^1^ The 17 targets—adopted by the United Nations member states in 2015—have been continuously monitored to ensure proper allocation of resources and enactment of policies necessary to stay on track.^2^ In relation to SDG 6—availability and sustainable management of water and sanitation for all—efforts need to increase 4-fold.^3^ High-income countries—including the United States (US)—are not an exception; emerging evidence indicates that communities across these countries still have persistent gaps in adequate wastewater management.^4−9^ In the US, for example, more than 2 million households lack access to safely managed wastewater infrastructure.^10^

Such inadequate wastewater infrastructure management is especially exacerbated in underserved communities. These communities are established homes—not new developments—where:^8^ (1) households have limited (or lack thereof) sewer access (for reasons such as no available service or cost); *and* (2) appropriate onsite wastewater treatment systems (OWTS) are in failing condition (e.g., due to impermeable soils, high groundwater tables, topology), or inaccessible for most households (e.g., due to affordability issues). Among these vulnerable underserved communities are small, rural communities that experience extreme operating conditions, including remote setting (being far from existing sewer system), low population density (small customer base), limited financial capacity (affordability issues), and limited local government capacity (e.g., staff, expertise, political resources).^8,11^ The focus of this study is these small, rural, underserved communities with such extreme conditions—referred to as *“rural, low-resourced communities”* hereafter for short. Notably, poverty rates across the US are not necessarily higher in all rural communities; some rural areas have higher median household incomes.^12^ However, throughout the US, over 60 million residents live in rural, low-resourced communities,^13^ which have long struggled to access and properly maintain functioning, reliable, and affordable wastewater infrastructure.^7^ Unfortunately, some of these communities use improper methods for wastewater disposal—including failing septic systems and straight pipes (defined as a direct discharge of raw sewage to the ground surface or streams^14^). For instance, over 60 thousand straight pipes discharge an estimated 6.75 million gallons of raw sewage into Minnesota waters every day.^14^ Similar issues have been experienced in rural communities in Alabama,^6,15,16^ Appalachia,^17,18^ Florida,^19,20^ central Michigan,^21^ Long Island, NY,^22^ Hampton Roads, VA,^23^ and Alaska.^24^ Such growing issues with failing wastewater infrastructures and associated adverse public and environmental health effects have driven commitment from the US Federal Government—through recent US federal infrastructure funding^25,26^—to prioritize addressing wastewater issues in rural, low-resourced communities.^27^ In this regard, the implementation of the Bipartisan Infrastructure Law (BIL)^26^ and American Rescue Plan (ARPA)^25^ has enabled wastewater systems access to funding through grants or fully forgivable loans. BIL provides $11.7 billion for Clean Water State Revolving Fund (CWSRF) through 2026,^28^ with 49% of those funds being grants to disadvantaged communities (e.g., low-income communities, rural areas, communities of color).^29^

Coupled with these unprecedented capital funding opportunities, scholars^30,31^ have been exploring alternative wastewater treatment and disposal strategies that consider rural, low-resourced communities’ unique needs and challenging operating conditions. Decentralized wastewater systems (DWS) have been demonstrated as effective solutions, assuring appropriate service levels and affordability.^30−33^ These decentralized systems—such as individual OWTS and clustered systems—collect, treat and reuse/dispose treated wastewater at or near the generation point;^11^ noting that a clustered wastewater system serves multiple dwellings (e.g., 100+ homes) that share a collection/treatment system (major typologies of DWS are further discussed in Figure S1 in the SI). What is still lacking, though, is an explicit conceptualization on how to best manage these small systems to ensure their adequate performance, reliability, sustainability, and affordability. To effectively manage such systems, a responsible management entity (RME) is often required to hold permits and conduct the operations and maintenance (O&M) services^34−36^—referred hereafter to as *responsible management*; with the RME being defined as a legal organization with the technical, managerial, and financial capacity to operate and maintain viable DWS within the RME’s jurisdiction and in accordance with appropriate regulations^34^ (refer to Figure S2 and Table S1 in the SI for the existing management framework of DWS and the life-cycle series of activities typically performed by the RME, respectively). It is important to note that the responsible management of rural DWS is a complex management model,^37^ as it is a context-specific process, designed in accordance with community’s resources and highly dependent on the cooperation across various community stakeholders.^11,34,38^ Additionally, RMEs’ financial sustainability is constrained by the extreme operating conditions in rural, low-resourced communities (e.g., limited number of rate payers, affordability issues). Accordingly, RMEs are often reluctant and disincentivized to consider managing small DWS. This makes these small systems vulnerable to inadequate O&M, challenging their ability to provide the level of treatment necessary to adequately protect public health and water quality.^36^ It is thus imperative to understand determinants that may influence the consideration of RMEs to provide O&M services to DWS in rural, low-resourced communities—referred hereafter to as *RME consideration*. Failing to do so introduces a gap in knowledge and practice regarding how to define and operationalize adequate RMEs that consider managing the DWS in these communities.

Indeed, there is a dearth in the academic literature examining the responsible management of DWS for achieving sustainable O&M services. Existing work is primarily published in agency reports,^34,36,39−41^ with a few exceptions of peer-reviewed articles^11,42^ and conference proceedings.^43−45^ Much of this work— published more than a decade ago—focuses on high-level characterization of general attributes surrounding the operation of RME, lacking any in-depth assessment of RME consideration for proper O&M. These attributes primarily fall under two dimensions—RME structure (e.g., viable types of entities)^11,36,41−44,46^ and operational aspects (e.g., system size).^34,36,41,44,46^ For instance, various types of RMEs could be considered for handling the O&M of DWS, including public and private service providers (e.g., water and wastewater utilities), as well as nonprofit corporations.^34,39,44^ Here, we posit that RME consideration is also shaped by barriers that may be faced by RMEs. In fact, decentralized wastewater infrastructure management is affected by numerous barriers,^11,39,41,43^ which span the technical, financial, social, institutional/regulatory, and environmental perspectives within which these systems operate^47,48^—referred hereafter to as *socio-technical barriers*. Such barriers, for instance, include limited technical expertise in regard to DWS (within the technical dimension); limited communities’ financial capacity to pay for O&M (financial); inflexible regulatory codes (regulatory/institutional); and lack of communities’ awareness to the adverse public health and environmental effects of inadequate wastewater management (social). Accordingly, this study proposes integrating into a single model the three dimensions of RME structure, operational aspects, and socio-technical barriers, more comprehensively capturing the conceptualization domain of RME consideration through a socio-technical systems lens. Operationally, pinpointing the most influential determinants would guide the establishment of a viable RME model for meeting the decentralized system’s O&M needs in the context of analysis. From this perspective, uncovering impactful socio-technical barriers could inform whether any changes in policy are proactively needed to overcome the concerning barriers, thereby contributing to operationalizing adequate RMEs for providing more sustainable O&M services that align with communities’ capacity and needs.

The proposed RME consideration approach seeks to answer the following question: In rural, low-resourced communities, what are the impacts of socio-technical barriers on entities’ consideration to provide O&M services to DWS, accounting for RME structure and operational aspects? To answer this question, the objective of this study is 2-fold. First, the study explores the primary determinants of RME consideration—spanning the three dimensions of RME structure, operational aspects, and socio-technical barriers—by turning to literature related to decentralized wastewater infrastructure management. Second, using the rural Alabama Black Belt as an example context, the study empirically assesses the impacts of the identified determinants on the provision of O&M services to DWS in these communities.

Using binomial logistic regression modeling of survey data from 114 public and private management entities spanning 27 states in the US, this study analyzes these entities’ consideration to manage alternative DWS for addressing wastewater issues in the Black Belt. This empirical assessment provides a holistic understanding of the operation of RMEs in rural, low-resourced communities, such as the Black Belt. Further, our discussion addresses the recent President’s National Infrastructure Academy Council’s (NIAC) call^49^ in providing low-income communities access to safely managed water-wastewater systems. While the analysis primarily focuses on the Black Belt, the proposed RME consideration approach could be implemented in other rural, low-resourced communities (e.g., rural Alaska, Appalachia, Texas colonias, and Navajo Nation). Though these communities are recognized to have commonalities (e.g., extreme geological conditions, high poverty, low-population density), specific communities may generate somewhat different findings, primarily shaped by their unique operating environments (e.g., local histories, socio-technical challenges). The applicability of the proposed RME consideration approach could also be extended to suburban contexts for managing small satellite treatment plants and decentralized systems. Additionally, the proposed approach could inform efforts for operationalizing RMEs in other decentralized infrastructure sectors (e.g., electricity, solid waste, water, cellular, and Internet management).

## 2 Materials and Methods

### 2.1 Identification of Determinants of RME Consideration

To identify the various determinants that may influence RME consideration, we turned to literature related to the management models specific for handling O&M of onsite and clustered wastewater systems (e.g., US-EPA^50^), as well as the wider literature related to decentralized wastewater management (e.g., Pinkham et al.,^39^ US-EPA^41^). Before conducting the literature review, we developed a list of search terms based on the scope of the study, considering system typology (i.e., decentralized, decentralised, cluster, clustered, shared, networked, distributed, onsite, on-site, “on site”, septic, nongrid, non-grid, “non grid”, “remote area”); water-sector management field (i.e., wastewater, “waste water”, sanitation); type of treatment (i.e., treatment, collection, disposal); management model/structure (i.e., responsible management entity/entities, RME, management, operation, operations, maintenance, O&M); and spatial setting (i.e., rural, non-urban, “non urban”). We applied these search terms to both the Web of Science and Scopus databases, yielding very few peer-reviewed journal articles that discussed responsible management of DWS in rural areas— further re-emphasizing the significance of this study in addressing this research need. Given such a dearth of academic literature, we also considered agency reports (e.g., EPA reports) and conference proceedings; the relevant studies and reports are cited and discussed in the Results section. The identified set of primary determinants of RME consideration was validated by six subject-matter experts (SMEs) to ensure its comprehensiveness; these experts have decades of experience in decentralized wastewater management in small communities. Notably, limited technical expertise exists on decentralized wastewater management and responsible management of DWS, and as such capturing these SMEs’ insights into the validity of the results from the literature review has strengthened the contribution of this work. The validated determinants—spanning the three dimensions of socio-technical barriers, RME structure, and operational aspects—informed the development of the survey questionnaire through including questions that target each of these determinants; further details are included in the Structure of the Survey Questionnaire section.

### 2.2 Study Site

Historically, the Black Belt region of Alabama—comprised of 17 out of Alabama’s 67 counties^51^—has struggled from a lack of access to safely managed wastewater infrastructure.^31,52−56^ In addition to the region being characterized by low-population density, rural poverty, and lack of economic development, the geological conditions (soils) have further exacerbated the wastewater crisis in these underserved communities.^6,31,52,57^ The shrink-swell clays (vertisols)—the most common surface soils in this region— become practically impermeable when wet; this soil condition prevents infiltration of effluent into the ground and consequently causes hydraulic failure in conventional OWTS (e.g., septic tank drainfield).^31^ In Bibb County Alabama, for instance, a field survey of 2000 unsewered homes found that around 50% had raw sewage on the ground surface due to the hydraulic failure of their septic systems, as well as the use of straight pipes for a direct raw sewage discharge.^54^ Unfortunately, the percentage of people who are still without proper access to wastewater services ranges between 50% and 85% in eight of the Black Belt counties (e.g., Perry, Sumter, Lowndes, Macon, Butler); refer to Table S2 in the SI for further details. Such unacceptable sanitation conditions have drawn both national and international attention^6,53,58−60^ and spurred research efforts to explore alternative wastewater treatment technologies, management models, and regulatory actions that can address wastewater issues in the Black Belt.^31^

Given widespread poverty,^54^ advanced OWTS for these soil conditions are not financially feasible in this rural region.^6^ As potential technological solutions, ongoing research efforts^31,33,61^ are exploring simpler onsite treatment options and effluent sewer and treatment clusters (e.g., 90+ homes), suitable for clustered unincorporated communities in the Black Belt. What is still needed, though, is a deeper understanding of how to provide O&M services that can ensure these systems’ long-term sustainability. This involves defining and operationalizing adequate RMEs that consider providing O&M services for these decentralized infrastructures, serving as a promising case to demonstrate our proposed RME consideration approach—i.e., exploring primary determinants of RME consideration and assessing their impacts on the provision of adequate O&M services.

It is important to note that most of the incorporated communities in the Black Belt counties have a limited number of wastewater connections (households served) and, in fact, are similar in size (number of connections) to many of the proposed decentralized clustered systems in unincorporated areas. Currently, about 50% of the Black Belt population is served by sewer (within incorporated communities), while the proposed decentralized clusters could potentially provide sewer access to another 30% of the population. For instance, Hayneville—the county seat of government in Lowndes County—has only 255 connections (see Table S2 in the SI). As such, small municipal systems might be considered one of many decentralized infrastructures applicable for centralized responsible management, in addition to the many proposed/recently installed clustered and onsite systems.

### 2.3 Structure of the Survey Questionnaire

A survey questionnaire was developed to assess the impacts of socio-technical barriers on the O&M of DWS. The survey included 51 questions, of which eight questions were related to the participants’ demographics and experience in their respective organizations. The remaining questions cover topics related to the entity type and structure (e.g., public service provider, private agencies, nonprofit), type of service provided (e.g., water, sewer), whether the entity currently operates small DWS, the entity’s potential consideration to operate and maintain new DWS, and a set of major barriers to effective decentralized wastewater management spanning the technical, financial, regulatory/institutional, and social/environmental dimensions. Participants were asked to specify if these are possible reasons that may prevent them from operating and managing such DWS, and accordingly rate their concerns based on a 5-point Likert scale (very concerning, concerning, neutral, somewhat concerning, not concerning). Table S3(a) in the SI shows specific questions of interest related to variables used to develop the BL regression model for assessing the RME consideration. Notably, this study is part of a larger practice-driven research project focusing on investigating cost-effective decentralized wastewater treatment technologies and management approaches that could effectively guide the implementation of alternative DWS in rural, low-resourced communities, like the Black Belt. The survey questionnaire covered other questions related to the treatment technological aspects—not included in Table S3(a) because it was out of this study scope. Examples of such questions include the average flow of clustered treatment systems that may be managed by the surveyed entity, types of wastewater collection systems used (e.g., gravity, pressure, STEP), number of lift stations employed, types of wastewater treatment technologies used (e.g., lagoons, recirculating media filter, aerated), and fate of treated effluent (e.g., surface discharge, drip-irrigation).

In addition to the intellectual motivation and need of this study, findings of this work have been sought to inform our on-the-ground efforts for identifying potential RMEs who are willing to provide O&M services to newly installed DWS—clustered and onsite—in the Black Belt. Therefore, we framed the survey question related to the RME consideration as a binary variable (see Table S3), aiming to receive a definite answer (i.e., Yes—considering; No—not considering). However, we acknowledge that RMEs may occupy a spectrum of dispositions around managing DWS, often shaped by a range of motivations, capacities, limiting barriers, and local contexts. As such, surveying the RME consideration as a categorical question (e.g., using a 5-point Likert scale) would better capture such a wider spectrum. This could be an interesting future research avenue toward advancing the modeling of RME consideration for the provision of successful O&M services to DWS in rural, low-resourced communities.

To ensure clarity, a short, recorded presentation was attached to the survey, providing an overview of the proposed alternative DWS for the Black Belt, technologies used, and the purpose of the survey. Additionally, prior to survey deployment, the content was validated through content review by 5 SMEs. Their expertise spanned DWS design and management, survey analysis, and public communication. The survey questionnaire was designed using Google Forms platform^62^ and was reviewed and approved by the Institutional Review Board at The University of South Alabama. Notably, given that the study captures the survey participants’ technical expertise rather than their personal opinion, it was determined as “exempt”.

### 2.4 National Survey Deployment and Data Collection

Based on the process paradigm of centralized management of DWS (i.e., RME managing multiple existing systems by utilizing SCADA or other remote monitoring devices, while acquiring required expertise and operational licenses), we targeted entities operating across various states in the US, as well as various types of entities (even nonwastewater focused). The survey was deployed to small-, medium-, and large-sized public and private entities (e.g., water utilities, sewer utilities, nonprofit organizations, community development corporations). Given that our objective is to capture entities’ consideration to serving as RMEs in the Black Belt, we targeted a single response per entity; responses were sought from professionals with executive, decision-making, or supervisory roles. To identify potential survey participants, we utilized random sampling,^63^ convenience sampling,^64^ and snowball sampling^65^ methods. In this respect, through online searches, we randomly identified a list of potential participating entities spanning different states. This list was then narrowed down to primarily consider entities where e-mail addresses of potential survey participants are provided on their Web sites. Additionally, we attended and presented at a local water-wastewater sector industry conference that attracts representatives from public and private utilities. During the presentation, discussions yielded insightful feedback from attendees with regard to barriers to effective decentralized wastewater management and establishment of RMEs. Attendees also helped establish connections with additional potential entities that operate across various states (i.e., snowball sampling). As part of snowball sampling, we also included a question in the survey, asking respondents to recommend entities (and their contact information) that may be adequate for managing small DWS in the Black Belt. Moreover, we leveraged our professional networks to identify additional potential survey participants (i.e., convenience sampling). These sampling efforts yielded an initial population of 725 potential participants. Given that we utilized the random sampling through online searches, we wanted to ensure that the survey is sent to a clearly defined and refined population.^66^ To do so, we first precontacted these potential participants individually using customized emails that highlight research objectives, participants’ roles, and how their experiences would inform our respective research efforts. Coupled with multiple reminders using phone calls or emails, 161 potential participants confirmed receipt and respective expertise, to whom the survey was delivered. The majority of these 161 participants who responded to our initial contact have expertise in wastewater infrastructure systems and provide wastewater services as part of their regular operations (e.g., wastewater-only, water-wastewater), with relatively lower response from those who do not serve wastewater systems (e.g., water-only, electricity-only, gas-only).

Data collection process—involving sampling, precontacting participants, survey delivery, and survey completion—was conducted between March 2022 and January 2023; notably, the first and last survey responses were received on March 31, 2022, and January 17, 2023, respectively. All respondents participated voluntarily and were over 18 years old. The final sample consisted of 121 valid responses from 121 management entities that span 27 states (see Table S4 in the SI), achieving a high response rate of 75%. Respondents included utility directors, executive directors, presidents, vicepresidents, founders, and wastewater specialists, with 59% having more than 20 years of experience. Additionally, 80% of the responses were received from public service providers (e.g., water and wastewater utilities). For more details about the respondents’ demographics, refer to Figure S3 in the SI.

### 2.5 Regression Analysis: Binomial Logistic Modeling

Prior to model development, we examined correlations in the predictors using the correlation matrix,^67^ as well as the Variance Inflation Factor (VIF),^68^ to determine any possible multicollinearity across independent and control variables. In this regard, correlations that are less than 0.5 indicate no collinearity issues.^67^ As for VIF, values that are less than 5 indicate a low correlation of that predictor with other predictors (i.e., no collinearity issue), while VIF values between 5 and 10 indicate a moderate, yet an acceptable correlation.^68^ Given that the dependent variable (i.e., “RME consideration”) is framed as a dichotomous variable (with values 0 and 1), we applied BL regression.^69^ A BL regression model predicts the probability that an outcome falls into one of the two categories of a dichotomous dependent variable based on predictors. The coefficient estimates for a BL model represents the log odds of an outcome, noting that the odds are equivalent to the ratio of two probabilities, specifically the probability of the outcome occurring to that not occurring
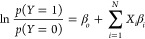
1where *N* is the number of predictors, *Y* is the observed RME consideration, *X* is the vector of predictors (i.e., independent variables: socio-technical barriers; control variables: RME structure and operational aspects), *β*_*o*_is the intercept, and β is the vector of estimated parameters (i.e., log odds of the outcome). Notably, the *odds ratios*, along with their confidence interval (CI), are commonly used to interpret the effects of predictors on the dependent variable; see eq 2:
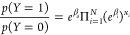
2where *e*^*β*_o_^ represents the odds of the outcome where all inputs are zero, and *e*^*β*_i_^ represents the odds ratios associated with a unit increase in *x*_*i*_ assuming no change in the other inputs (i.e., a unit increase in *x*_*i*_ multiples the odds of an outcome by *e*^*β*_i_^). Notably, an odds ratio greater than 1 corresponds to “positive

effects” because it increases the odds of the outcome occurring (compared to nonoccurrence).^69^ On the other hand, an odds ratio between 0 and 1 corresponds to “negative effects” because it decreases the odds of the outcome occurring (compared to nonoccurrence).^69^ Odds ratios of exactly 1 correspond to “no association”, and an odds ratio cannot be less than 0.^69^

To assess model fit, we used McFadden’s *pseudo-R*^*2*^ measure, with values above 0.2 generally indicating a good model fit.^70−73^ Notably, we performed all statistical analyses using *R* software^74^ and various supporting packages, including *tidyverse*(75) (used for data import, tidying, manipulation, and data visualization), *gplots*(76) (v3.1.1; used for plotting data), *lmtest*(77) (v0.9–40; used for developing the BL regression model), *corpcor*(78) (v1.6.9; used for testing correlations), and performance^79^ (used for evaluating the performance of the BL regression model using *pseudo-R*^*2*^ measure).

### 2.6 Data Processing

Table 1 shows the independent and control variables that are incorporated into the BL regression RME consideration model. Prior to data analysis, we examined the data completeness of the entire data set; this yielded 114 complete responses that were used for model development. This sample size (greater than 100) is considered sufficient to reduce bias in BL regression model estimates.^80,81^ To further improve the reliability of parameter estimates and interpretation of results,^69^ we recoded the categorical independent and control variables to be binary—except for management scale and system size determinants (see Table 1). For instance, for the socio-technical barriers, “very concerning” and “concerning” answers were recoded to “1”, whereas all other answers were recoded to “0”. Similarly for the entity type, “1” was assigned to the “public” answer and “0” to others (e.g., private, nonprofit). For the service type, “1” indicates that the wastewater service is provided by the entity (e.g., wastewater-only, water-wastewater, hybrid, including wastewater), whereas “0” denotes otherwise. The location of operation is recoded to “0” indicating that the entity operates in a location different from the proposed DWS (i.e., outside Alabama), and “1” indicating the same location (i.e., the entity operates in Alabama). The management scale determinant has four categorical levels, 1 to 4, denoting community, county, regional, and state, respectively. Similarly, for the system size, “1” to “4” categorical levels denote small, medium, large, and very large, respectively. The remaining determinants are binary, with yes/no categorical levels. For further details related to the answers to each survey question and corresponding recoded categorical levels used for BL regression modeling, refer to Table S3 in the SI.

**Table 1:** Variables and corresponding Recoded Categorical Levels Used for BL Regression Modeling

Variable	Recoded categorical level
**Independent variables: Socio-technical barriers**	
*Operator turnover*	0–Not concerning; 1–Concerning
*Financial incentives*	
*Public funds*	
*Financial capacity*	
*Operational cost*	
*Regulatory codes*	
*Liability concerns*	
*Organizational structures*	
*Environmental awareness*	
*Equity concerns*	
**Control variables: RME structure and operational aspects**	
*Entity type*	0–Nonpublic; 1–Public
*Management scale*	1–Community; 2–County; 3–Regional; 4–State
*Service type*	0–Wastewater not provided; 1–Wastewater provided
*System size*	1–Small; 2–Medium; 3–Large; 4–Very large
*Location of operation*	0–Different; 1–Same
*Decentralized service operation*	0–No; 1–Yes
*Centralized service operation*	0–No; 1–Yes
*Operational flexibility*	0–No; 1–Yes

## 3 Results

### 3.1 Determinants of RME Consideration

As shown in Table 2, the determinants that may influence RME consideration are categorized into RME structure, operational aspects, and socio-technical barriers. For instance, within the social barriers, the potential increase in property taxes often associated with improved access to basic sewer services in the community areas may cause affordability issues to these rural communities.^39^ Such affordability issues, in turn, may lead to potential change in the community socio-demographics (due to reasons such as gentrification) and raises the concerns of not meeting the actual underserved communities’ needs^82^—referred to here as equity concerns. RMEs may not want to deal with such community-related concerns, especially because articulating them in terms of local conditions may be politically divisive.

**Table 2:** Primary Possible Determinants of RME Consideration, Identified from Literature

Dimension/determinant	Explanation/reference
**Socio-technical barriers**	
*Technical*	
High operator turnover and limited technical expertise	• Difficulty retaining skilled operators due to high turnover in rural areas,^43^ leading to limited technical expertise in the O&M of DWS.^39,41,43,44^
*Financial*	
Limited financial incentives	• Limited to no financial incentives to manage new DWS.^41,43^
Difficulty to obtain funds	• Difficulty obtainig public funds/capital for privately owned or managed systems.^41,43^
Limited financial capacity to pay for O&M services	• Limited communities’ financial capacity to pay for the O&M of DWS.^39,43^
Unclear operational cost	• Unclear operational cost of DWS.^39^
*Regulatory/institutional*	
Inflexible regulatory codes	• Inflexible and prescriptive regulatory codes that may hinder the inclusion of new nonconventional systems or the operation of systems outside the service area.^39,41^
Liability concerns	• RMEs’ concerns from potential liability and/or risk of regulatory noncompliance associated with managing unfamiliar systems and possible consequential system failures.^39,49^
Lack of organizational structures	• Lack of necessary organizational structures for effectively managing DWS.^39,41^
*Social/environmental*	
Lack of awareness to consequences of failing systems	• Lack of communities’ awareness to possible environmental and public health risks associated with failing wastewater systems, which may impact their willingness to pay for the O&M.^39,41^
Equity concerns	• Potential change in community socio-demographics and concerns of not meeting community actual needs;^82^ e.g., due to potential increase in property taxes often associated with improved access to basic services in the community areas.^39^
**RME structure**	
Entity type	• Several types of organizations could serve as RMEs, including public, private, and nonprofit.^34,39^
	• These entities’ consideration to manage small decentralized systems may be influenced by their characteristics (e.g., type) and their legal status.^34,39,44^
Management scale	• O&M of DWS can be performed at various management scales, depending on the RME’s jurisdiction,^34^ including community-level, county-level, across multiple counties (regionalization), and state-level.
	• The management scale may impact entities’ consideration to provide O&M to DWS.
	• Regionalization of the responsible management of small DWS may be preferred by RMEs to ensure financial sustainability.^49,83−86^
	• RMEs may not consider managing systems across various jurisdictions (e.g., counties), due to reasons such as service area restrictions^39,41^ or political dynamics.^86^
Service type	• These entities’ consideration to manage small decentralized systems may be influenced by the services they provide, such as wastewater-only, water-only, water-wastewater.^38^
	• For example, entities providing wastewater services may be more willing to serve DWS than those not operating in that space.
**Operational aspects**	
System size	• Defined by the number of equivalent dwelling units (EDUs).
	• The system size influences the willingness of existing RMEs to consider managing new alternative decentralized systems.^34,36^
	• As the number of EDUs increase, RMEs may be more willing to consider serving the DWS, as this would help with maintaining greater customer base and financial sustainability.^34^
Location of operation	• RMEs’ existing operations’ location may impact their consideration to serve new systems in other areas (e.g., different states or counties), as RMEs may (or may not) be willing to use remote monitoring and control needed for operator efficiency and performance tracking when adopting centralized management approach of DWS.^34^
	• Nonadoption of remote monitoring may be due to financial obligations of its technical requirements, e.g., software and hardware requirements, data acquisition, transmission, storage, and analysis.^11,87^
Decentralized service operation	• Whether RMEs currently provide O&M services to DWS or not.
	• Providing decentralized services may increase existing RMEs’ consideration to serve new decentralized systems, as they would have the required technical expertise and licenses needed to perform O&M.
	• Otherwise, RMEs should be willing to obtain these technical requirements to fulfill system compliance to operating permits.^34,35^
Centralized service operation	• Whether RMEs currently provide O&M services to centralized wastewater systems or not.
	• RMEs only providing centralized wastewater service may not consider managing decentralized systems, as this requires expertise and licenses that may not be available in-house.
	• As previously noted, RMEs should be willing to obtain these technical requirements to fulfill system compliance to operating permits.^34,35^
Operational flexibility	• Defined by RMEs being able to operate outside their service area (i.e., not having service area restrictions) and/or accept effluent from nearby communities.
	• Such operational flexibility is typically constrained by the regulatory codes in the context of DWS operations.^39,41^
	• Operational flexibility may impact RME consideration, as RMEs’ service area restrictions may hinder their ability to provide services to systems outside their service area.

### 3.2 Descriptive Statistics

Over 70% of entities that participated in the survey provide wastewater services along with at least one other service (e.g., water, gas, electricity, and solid waste), whereas 27% of entities only serve wastewater systems (see Figure S4 in the SI). Additionally, the distribution of responses across the system size categories (i.e., small, medium, large, very large) is almost equal; refer to Figure S4 for further details about the distribution of responses by the various control variables (e.g., management scale, operational flexibility).

Several independent and control variables exhibited collinearity issues (correlations greater than 0.5), including “financial incentives”, “public funds”, “operator turnover”, “organizational structures”, “regulatory codes”, “liability concerns”, “service type”, and “centralized service operation” variables (see Table S5 in the SI). To resolve such issues, we excluded “financial incentives”, “organizational structures”, “service type”, and “centralized service operation”. The VIF values of the final generated RME consideration model indicated no collinearity concerns; values are all below 5 (low correlation) (Table S6 in the SI).

### 3.3 BL Regression RME Consideration Model

Table 3 summarizes the results for the odds ratios of the RME consideration model; refer to Table S7 in the SI for the BL regression analysis results. The relationship between the “RME consideration” and “operator turnover” is statistically significant at the 1% significance level. Additionally, the relationships with the “entity type”, “management scale *–* county”, “decentralized service operation”, “operational flexibility”, and “regulator codes” are statistically significant at the 5% significance level (see Table 3). Regarding determinants of categorical data type, it should be noted that the BL regression model assesses parameter estimates for the levels of the categorical variables relative to a reference level. Given that the variables in the RME consideration model are all binary—except for “management scale” and “system size” (see Table 1)—the parameter estimates of the level “1” (Table S7) and their corresponding odds ratios (Table 3) are relative to level “0”. Regarding the “management scale” variable, the estimates of its three levels—“county”, “regional”, and state—are relative to the “community” level. Similarly, the estimates of the “medium”, “large”, and “very large” levels related to the “system size” variable are relative to those of the “small” level.

**Table 3:** Odds Ratio Results for the RME Consideration Model^a^

Variable	Odds ratio^b^	2.5%	97.5%	*p*
*intercept*	0.08	0.00	1.98	0.16
**Independent variables: Socio-technical barriers**				
*Operator turnover*	0.13	0.03	0.49	0.004***
*Financial capacity*	2.55	0.47	17.4	0.30
*Public funds*	0.77	0.19	2.95	0.69
*Operational cost*	0.49	0.11	2.04	0.03
*Regulatory codes*	4.62	1.24	20.02	0.03**
*Liability concerns*	1.59	0.38	6.81	0.52
*Environmental awareness*	0.65	0.18	2.25	0.49
*Equity concerns*	2.89	0.84	11.00	0.10
**Control variables: RME structure and operational aspects**				
*Entity type*	0.13	0.02	0.78	0.03**
*Management scale–county*	6.16	1.36	34.75	0.02**
*Management scale–regional*	2.37	0.42	14.36	0.33
*Management scale–state*	4.27	0.29	62.19	0.28
*System size–medium*	0.67	0.13	3.91	0.64
*System size–large*	0.75	0.12	4.8	0.75
*System size–very large*	0.35	0.05	2.42	0.28
*Location of operation*	1.95	0.58	6.84	2.86
*Decentralized service operation*	4.55	1.19	19.33	0.03**
*Operational flexibility*	11.44	1.55	264.47	0.04**

a BL regression analysis–odds ratios at 95% CI. *p < 0.1. **p < 0.05. ***p < 0.01.

b Odds ratio > 1 → odds of the outcome occurring is larger for the corresponding predictor’s categorical value compared to its reference level. 0 < Odds ratio < 1 → odds of the outcome occurring is lower for the corresponding predictor’s categorical value compared to its reference level.

As shown in Table 3, the values of the odds ratios either lie between 0 and 1 or are greater than 1. For instance, the odds ratio of the “entity type” variable is 0.13 (see Table 3), which corresponds to level “1” (i.e., public) as compared to level “0” (i.e., nonpublic). Given that the value lies between 0 and 1, it indicates that the odds of an entity considering managing DWS is 87% (i.e., 1–0.13 = 0.87; equivalent to 87%) *lower* for public entities compared to those that are nonpublic. On the other hand, the odds ratio of the “management scale-county” is 6.16; the fact that the value is higher than 1 indicates that the odds of the entity considering managing DWS is 6.16 *times higher* for an entity that supports county-level management compared to an entity that supports community-level management (i.e., reference level). Notably, the RME consideration model has a very good fit with McFadden’s *pseudo-R*^*2*^ measure of 0.36 (see Table S7).

## 4 Discussion

### 4.1 Impactful RME Structure and Operational Aspects to Effective Responsible Management

#### 4.1.1 Consideration of Privatization for Effective Entity Type

Results show that being a public entity reduces the odds of an entity considering providing O&M services by 87%, compared to being a nonpublic entity— an odds ratio of 0.13 (see Table 3); that is, a public entity is less willing to consider operating and managing small DWS. This important finding unveils a critical underlying challenge to successful management of alternative DWS, especially that over 80% of water-wastewater utilities in the US are publicly owned and operated by municipalities.^49^ The fact that public entities typically have defined jurisdictions for their service area (e.g., town/city, county) may be one of the barriers limiting their consideration to managing DWS in the Black Belt, especially because many of these communities are mostly unincorporated (i.e., outside city limits). On the other hand, private entities—being driven by profit and economic efficiency^88^—are typically more willing to expand their service jurisdiction to connect more households, further promoting their financial sustainability. From a socio-political perspective, public entities are typically more sensitive to politically based pressures, especially those involving public health concerns that could lead to politically costly public response.^89^ In fact, the Black Belt is a politically pressured context, with its acute wastewater problem receiving both national and international press attention.^53,60^ Local politicians and public entities in these rural, low-income communities are hesitant to mandate sewer hookups and the payment of monthly sewer fees, as this may result in negative political votes. While the Alabama Department of Public Health (ADPH) onsite sewage treatment and disposal rules^90^ mandate sewer hookup if an onsite system is failing, ADPH does not broadly enforce this rule—particularly in the Black Belt—due to political pressures. Such political dynamics may have disincentivized the surveyed public entities to consider managing the DWS in the Black Belt communities. These interesting analyses—linking entity type to service operation and political considerations—merit future research efforts to further investigate barriers specific to public entities, as well as provide a comprehensive discussion of their incentives (or lack thereof) to managing alternative DWS considering finances, profitability, and political dynamics.

Given that public entities are less inclined to deliver decentralized wastewater services in the Black Belt, findings suggest considering alternative entity types, such as private agencies (e.g., private utilities, community development corporations) and nonprofit organizations. Such findings align with the recent calls from the President’s NIAC^49^ to consider privatizing water-wastewater utilities for effective and sustainable systems operations and management. According to a public media article,^91^ proponents of privatization believe that changing ownership in the water-wastewater sector would alter incentives and accountability, while maintaining access to the private firms’ engineering and financial know-how that is much needed in light of a growing string of water-wastewater crises—especially exacerbated in rural, low-resourced communities.^49,91^ However, private entities are typically not eligible for federal funding, thereby challenging these entities to provide sustainable O&M service in rural, low-resourced communities that already struggle from poverty and low-population density. Even at the household level, direct loans to homeowners are uncommon because, under state statutes, many CWSRF programs are only able to make loans to public entities, such as municipalities, counties, state agencies, districts, or other political subdivisions.^27^ As such, to further support the urgent need for adequate wastewater management in rural, low-resourced communities, such as the case of the Black Belt, federal and state policy need to address gaps in funding alternative ownership models. For instance, policy changes are needed to allow access of privately owned water-wastewater providers to US Federal grant programs,^49^ thereby contributing to addressing barriers to privatization and other nontraditional ownership models in the water-wastewater sector.

While privatization seems promising to enable an effective utility structure for sustainable responsible management of DWS, Wells et al.^92^ highlighted the need to consider social (e.g., communities’ preferences) and political settings of local infrastructures to ensure equitable systems management and operations. For instance, communities’ residents may oppose privatization, due to concerns of experiencing higher rates and less transparency under private ownerships.^91^ While rates under privately owned systems are typically higher than those under their publicly owned counterparts, a recent study^93^ conducted in California interestingly reveals that regulated privately owned systems provide more low-income assistance than publicly owned systems. Even for the case of public entities, underserved communities often lack trust and confidence in these entities’ capabilities in providing financially sustainable O&M services. Such distrust is embedded in a long history of state, county, and local governments’ failures to address wastewater issues and provide sufficient O&M funding in these communities.^49^

Given such wrestling with this challenge of siloed entity structure—public versus private entity—a public-private partnership (PPP) may be an effective RME model option for managing alternative systems in critical areas with extreme operating environments (e.g., geological conditions, political dynamics),^49,94^ such as the case of the Black Belt. In such an emerging model, the local government would provide the legal authority to create the PPP arrangement and enforce it, as well as the ability to obtain federal and state grants and loans. The private RME, on the other hand, would design and build systems leveraging their technical and managerial efficiencies, as well as provide O&M services post-installation. Sheehan^94^ suggests establishing such a PPP arrangement via a special district; for instance, counties and municipalities may create these districts and assess fees for the services provided.

#### 4.1.2 Shared Responsible Management for Alternative Decentralized Wastewater Systems

The odds of an entity considering providing O&M is 6.16 times higher for an entity that believes county-level management as a reasonable scale jurisdiction, compared to an entity that supports community-level management (an odds ratio of 6.16; see Table 3). As such, to enable more sustainable operation in the Black Belt, results suggest establishing centralized responsible management of DWS within a county. For potential RMEs serving rural, low-resourced areas where residents are used to not paying for wastewater treatment, entities’ financial sustainability can be particularly concerning. Realistically, managing multiple clusters within a county (e.g., 600 to 4000 customers) would better support the financial sustainability of RMEs compared to operating at the community level (e.g., 200 to 300 customers), especially that such rural communities have low-population density to ensure reasonable utility rate base. Operating a larger pool of clusters, in turn, could enhance cost-effectiveness and service affordability for community members, as the O&M cost would be shared across a larger pool of customers within the county. Even the construction costs associated with installing collection and treatment systems are best lowered by spreading it among more users.^44^ For the case of the Black Belt, ongoing research efforts for determining decentralized wastewater clusters’ locations and sizes show that each county has between 600 to 4,000 new connections within multiple potential decentralized clustered systems. As such, increasing the scale of responsible management for these DWS would bring more cost-effectiveness to community residents as well as long-term financial sustainability to the RME.

In addition to sharing construction and operational costs, centralized management of DWS provides rural, low-resourced communities with an opportunity to share information and resources (e.g., equipment, treatment materials, operators) with their nearby communities through cooperative agreements.^35,95^ To ensure effective implementation of such management approach, communities need to (1) assess their existing regulatory structure to identify whether any changes in policy (e.g., service permitting) are proactively needed, and (2) consider the skills required to provide the targeted management scale.^38^ Additionally, RMEs managing alternative DWS at a larger scale (e.g., county-level) should develop a comprehensive plan for establishing rates for O&M services and handling billing and payments. For instance, implementing a hybrid payment approach—such as linking RME service fees with water, gas, cable, electricity, Internet bills or other service—can help ensure payment.^34^

Reports published by federal agencies^49,85^ and nonprofit organizations^83,84^ suggest that regionalization (defined here as managing DWS across multiple counties) even provide better opportunities for rural, low-resourced communities through (1) achieving economies of scales, operational efficiencies, and collective expertise; (2) accessing lower capital cost while maintaining customer rates; and (3) stabilizing revenue across a more diverse customer base. Despite the technical, financial, and managerial advantages of regionalization of management, community preferences and political dynamics across communities must be considered.^92^ For instance, regionalization decisions—also referred to as utility consolidation—can be politically fraught and risk opposition from communities involved with such agreements.^96,97^ Such political dynamics—embedded across counties in the Black Belt region^86^—might be one of the reasons why survey participants would not consider managing alternative DWS across multiple counties. Bakchan et al.^86^ and Elliott et al.^8^ found that such political resistance to regionalization is primarily due to insufficient education about the opportunities of regionalization with all types of stakeholders, including community residents, elected officials, regulators, and utilities. As such, community outreach and consultation are essential at each step of the decision-making processes related to establishing an appropriate management scale, further supported by educational efforts. Such efforts would achieve equitability in decision making for community residents, opportunities for codesign of their wastewater systems to respect their interests, and informed control over how to manage their systems.^8^

To further support and incentivize the regionalization of the responsible management of alternative DWS in rural, low-resourced communities, state capacity development regulations should be modified to ensure beneficial regionalization. For instance, NIAC^49^ suggests offering “safe harbor” from regulatory penalties to systems that absorb other troubled systems for a reasonable time period. Additionally, state and county legislation is needed to incentivize and/or mandate more regional RMEs in these communities, such as the recent legislation^98^ passed in New Mexico, mandating the consolidation of water-sector authorities to form economies of scale and strengthen rural water-wastewater systems operations. Changes in federal and state funding policies are also needed, such as modifying current grant allocation formulas to actively promote beneficial consolidation of water systems.^49^ More importantly, utility consolidation/regionalization needs to be undertaken proactively, with the early engagement of community leaders and stakeholders for evaluating options and determining appropriate institutional arrangements at the local level.^99^

#### 4.1.3 Consideration of RMEs’ Operational Flexibility and Decentralized Service Operation

Results, unsurprisingly, show that the odds of considering providing O&M services is 11.44 times higher for an entity that has operational flexibility compared to that with operational restrictions (an odds ratio of 11.44; see Table 3). Therefore, when identifying potential RMEs to provide O&M services in the Black Belt— whether considering existing entities or establishing new ones—it is important to ensure that RMEs can legally operate DWS outside their typical service areas and/or have the technical treatment capacity to accept effluent from nearby communities. Such operational flexibility is essential when implementing centralized responsible management approach for alternative DWS beyond the local scale (e.g., county-level, regionalization across multiple counties) to ensure meeting appropriate regulatory requirements (e.g., service permitting). Therefore, we recommend that local governments allow operational flexibility outside entities’ typical service areas, especially for regional RMEs that consider managing multiple DWS across a larger scale which goes beyond typical jurisdictional classifications (e.g., city, county). Such regional and flexible operations would assist rural, low-resourced communities—such as the Black Belt—that lack local governmental capacity (e.g., staff, expertise, finances) to maintain sustainable systems management.

Similarly, results show that the odds of an entity considering providing O&M services is 4.55 times higher for an entity that already operates and manages DWS, compared to the entity that do not provide decentralized wastewater management services (an odds ratio of 4.55; see Table 3). Unsurprisingly, having the required expertise and licenses already in place would likely increase the entity’s consideration to provide O&M services to other decentralized systems. As such, to increase the odds of successful operation in the Black Belt, it is suggested to consider RMEs that have decentralized wastewater management as part of their service operation. Otherwise, RMEs should be willing to obtain the required technical expertise and skills needed to perform O&M activities and fulfill system compliance to operating permits. For instance, gravity flow soil-infiltration systems require little O&M compared to systems employing advanced treatment technologies and electromechanical components that require more intensive O&M attention.^34,35^ Fortunately, management entities—even those outside the water-wastewater sector such as electric cooperatives—have recently started to change their mindset from viewing wastewater as a compliance cost to viewing it as a reliable and sustainable water source, as well as a business opportunity that could expand their business models.^49^ In spite of their willingness to expand their service operation to cover decentralized wastewater services, RMEs may be challenged by the limited operator trainings that provide this expertise in Alabama (further discussed below). Such training limitation may have disincentivized surveyed entities that do not provide decentralized wastewater management services to consider expanding their service to DWS.

### 4.2 Impactful Socio-technical Barriers to Effective Responsible Management

#### 4.2.1 Shortage in Certified Wastewater Operators, Challenging the Provision of Appropriate O&M

Results show that the technical barrier impacts the provision of appropriate O&M services to alternative DWS in rural, low-resourced communities. More specifically, the odds that an entity considers providing O&M services is 87% lower for entities that are concerned about the operator turnover barrier, compared to those that are not concerned about it (an odds ratio of 0.13; see Table 3). The decline in an adequately trained workforce in the water-wastewater sector is an ongoing national challenge, especially exacerbated in rural, low-resourced communities. Similar to the rural Alabama Black Belt, Spearing et al.^100^ confirms that operator turnover in rural Alaska, and the loss of institutional knowledge associated with that, make managing water and wastewater systems difficult. Other tribal communities (e.g., Navajo Nation) are also struggling from workforce challenges, with water-wastewater systems struggling to stay in compliance due to limited number of dedicated, certified operators.^101^ Well et al.^92^ confirms that the lack of certified wastewater operators in underserved communities is a major challenge that exacerbates the difficulty to provide sustainable O&M for small, alterative DWS. For instance, in Alabama, all permitted wastewater treatment facilities permitted by Alabama Department of Environmental Management (ADEM) must have a certified operator. Yet, operator training and certification do not exist for decentralized wastewater technologies that are more appropriate for rural, low-resourced communities, such as effluent sewers and recirculating media treatment systems often associated with lower O&M costs.

Survey respondents seem to be highly concerned about this workforce barrier in the Black Belt, as it would probably increase their hurdle to acquire required expertise needed to conduct timely O&M services and respond to issues as they arise. As such, addressing the operator turnover barrier would potentially increase the odds of entities considering providing O&M services to the proposed alternative DWS in the Black Belt. Novel mechanisms are needed to provide educational opportunities that address workforce shortage, such as developing hiring programs to train wastewater operators to get certified on appropriate decentralized technologies for rural, low-resourced communities, supported by continuous technical assistance and development. In Alabama, for instance, ADEM needs to upgrade their operator certification program to include training and certification on specific small-community collection/treatment technologies that are more cost-appropriate to onsite and clustered systems. Such training programs have been actually called for since more than a decade^11^ and continue to be much needed, exacerbating wastewater challenges in rural, low-resourced communities nationwide. NIAC^49^ has recently suggested additional investment in the water-wastewater infrastructure workforce by expanding fund programs—such as EPA’s Innovative Water Infrastructure Workforce Development Grant Program^102^—to train the next generation of water-wastewater facility operators. Further, additional efforts are needed to provide strategies for retaining staff and operators with valuable training and expertise in utilities within rural, low-resourced communities, such as providing competitive compensation as well as recruiting local workforce.^103^ As such, federal and state policy needs to prioritize funding such efforts, thereby addressing RMEs’ concerns to consider providing such services and ensuring continuous system operations and reliability.

#### 4.2.2 Regulatory Codes Not Aligned with the Local Settings of Infrastructures

BL regression results show that the odds that an entity considers providing O&M services is 4.63 times higher for entities that are concerned about the “regulatory codes” as an impactful barrier to effective decentralized wastewater management, compared to those that are not concerned about it (an odds ratio of 4.63; see Table 3). As such, addressing this barrier would likely contribute to providing effective O&M services to the proposed systems in the Black Belt. Decentralized wastewater management in the Black Belt is challenged by rigid and inflexible regulatory environment. DWS in this region must adhere to the same wastewater disposal regulations as the rest of Alabama despite the unique and extreme geological environment. It is important to note that, in Alabama, wastewater systems with an effluent flow rate of 15,000 gallons per day [GPD] or less—as well as discharge to the subsurface—are regulated by ADPH, whereas those with a flow rate above that threshold are regulated by ADEM. At the time of survey deployment, surface discharge of treated effluent from OWTS was still not yet permitted under ADPH regulations. Respondents may have been concerned about RMEs’ ability to obtain operating permits for these nonconventional DWS. Due to the challenging soil conditions for discharge in the Alabama Black Belt, recent changes (effective February 13, 2023) to ADPH codes have been approved for surface disposal from OWTS after treatment and disinfection, referred to as the Innovative Effluent Discharge System (Innovative EDS); this is approved for locations where traditional septic tank effluent will not infiltrate the clay soil.^104^ We encourage Alabama’s regulators to continue these efforts to update surface discharge-related regulations and consider a variety of treatment/disinfection systems that are cost-effective (e.g., both mechanical systems and more passive systems like constructed wetlands). These considerations would facilitate the attainment of the required permits and enable the RMEs to provide adequate services for the Black Belt communities. We also recommend establishing mechanisms to enable rural, low-resourced communities across the US to partner with each other to share information and best practices regarding decentralized wastewater management and corresponding regulatory actions, such as DigDeep’s Decentralized Wastewater Innovation Cohort^83^ that contributed to facilitating the recent regulatory updates in Alabama.

It is important to highlight that survey respondents may also be concerned from the rigid regulatory environment in Alabama due to its potential impact on their operational flexibility aspect. For instance, inflexible and prescriptive regulatory codes may prevent RMEs from operating systems outside their service areas, thereby limiting their consideration for the provision of O&M to alternative DWS in rural, low-resourced communities. Future research is, therefore, invited to further explore possible associations between regulatory codes and operational flexibility aspects.

#### 4.2.3 Social Equity and Financial Concerns Veiled by Availability of Public Funding

BL regression results (Table 3) show that the availability of public funds is not concerning to entities, probably due to current federal funding opportunities (e.g., CWSRF) prioritizing rural, underserved communities;^25,26^ even ADEM has allocated a significant portion of infrastructure funds to address historic wastewater infrastructure inequities in the Black Belt.^105^ However, it is important to highlight that these funds are not enough to cover all Black Belt communities. Additionally, these funding initiatives may introduce another layer of social inequity in these communities, knowing that infrastructure development may lead to potential increase in property taxes and consequential socio-demographic changes (for reasons such as gentrification due to affordability issues).^39^ Krings and Schusler^106^ referred to such a situation as environmental gentrification, which intentionally or unintentionally threatens the displacement of vulnerable residents. Lewartowska at al.^107^ added that such potential social inequity arising from infrastructure development—if not proactively addressed—may develop rejection from community residents toward implementing infrastructure solutions in their areas to resist the gentrification that tends to follow such development initiatives. To further explore possible relationships between funding initiatives and equity concerns, we excluded the “public funds” variable from the BL regression RME consideration model (see Table S8, Table S9, and Table S10 in the *SI*). Interestingly, the impact of “equity concerns” on the “RME consideration”—after the exclusion of “public funds”—becomes statistically significant at 10% significance level. This important finding sets the stage for future research efforts to further investigate possible unintended consequences of current federal and public infrastructure funding on social equity in rural, low-resourced communities. Additional efforts are needed to develop comprehensive strategies that drive direct benefits of DWS installation projects to existing residents, promote inclusive economic development, and proactively address unintended displacement consequences.

It is surprising that communities’ limited financial capacity is not an impactful barrier for survey respondents to consider providing O&M services (see Table 3). This unexpected finding may be attributed to a misconception surrounding the use of public infrastructure funds. Currently, most federal wastewater funding programs only provide capital/construction costs to communities, *not* O&M costs. However, over the operational lifetime of the system, the O&M costs are equally important to construction costs.^11^ Considering the financial limitations in rural, low-resourced communities, federal and state funds should be prioritized or program rules modified to in some way subsidize the O&M of DWS and meet their long-term maintenance needs. Besides, innovative financial mechanisms are needed to achieve financially self-sustainable O&M in these communities, such as proactively using appropriate system typologies (e.g., clustered systems) and regionalizing the responsible management to ensure sustainable customer base across communities. Additionally, public awareness relating to the extent of adverse health impacts as a result of improper sanitation is minimal in these communities,^11^ which may impact their willingness to pay for O&M. Therefore, environmental education as well as public awareness and participation—targeting all types of stakeholders—should be given high priority to achieve sustainable management of wastewater infrastructure. Providing local people with access to resources, education, and information necessary to influence environmental issues that affect them is a necessity. Appropriate wastewater infrastructure access, management, and affordability better protects public health and environmental health, as well as provides opportunities for economic development.

## 5 Outlook and Research Needs

The process of defining the responsible management of DWS through integrating the RME structure, operational aspects, and socio-technical barriers offers a powerful means to hypothesize about how to achieve successful RMEs’ operations in a specific set of rural communities—i.e., rural, low-resourced communities that experience extreme operating conditions, including remote setting, low-population density, and limited financial and local government capacity. In the ongoing effort to meet the long-term sustainable maintenance needs of DWS, this research presents an integrated approach for how future research may be conducted to address the theoretical complexity surrounding the RMEs in these communities. To the best of authors’ knowledge, this is the very first scholarly article to define RME consideration in rural, low-resourced communities, providing empirical understanding to the most feasible RME structure to handle the O&M services of DWS in the Black Belt, as well as the impacts of operational aspects and socio-technical barriers on RMEs’ consideration to provide these services. Our work also sets the stage to provide practical and policy recommendations that could best overcome the impactful socio-technical barriers. By addressing the wastewater infrastructure challenges in these underserved communities and achieving sustainable systems management, this research contributes to the global conversations on sustainable development. The applicability of the proposed RME consideration approach may also be extended to suburban contexts for managing small, satellite treatment plants and decentralized systems, as well as operationalizing RMEs in other decentralized infrastructure sectors (e.g., electricity, solid waste, water, cellular, Internet management).

Future research is invited to implement the proposed RME consideration approach in other rural, low-resourced communities (e.g., rural Alaska, Appalachia, Texas colonias, Navajo Nation) and explore how aspects of the BL regression RME consideration model of the Black Belt (Table 3) compare to those of other communities, given their different local histories, socio-technical contexts, and landscapes. Additional research is also called for to expand efforts toward the consideration of a diverse group of stakeholders (e.g., regulators, elected officials, engineers, utilities, community residents) for understanding socio-technical barriers to decentralized wastewater management, as well as challenges and opportunities underlying various RME structure options (e.g., public, private, PPP, county-level management, regionalization). Similar to the authors’ ongoing work, conducting semi-structured interviews to capture such stakeholders’ perceptions and their recent experiences with the infrastructure systems would (1) enhance the performance of the BL regression RME consideration model; (2) provide deeper insights into these stakeholders’ different perspectives on barriers specific to public entities, the privatization of ownership in water-wastewater sector, trade-offs between public and private models, as well as various aspects surrounding the establishment of PPP; and (3) enrich the limited scholarship related to the responsible management of DWS. Additionally, future research is needed to model the RME consideration as a categorical variable based on a 5-point Likert scale (e.g., not considering, somewhat considering, neutral, considering, strongly considering) to further strengthen the analysis through capturing a wider spectrum of RMEs’ dispositions to managing DWS. Further, exploring interaction terms in the BL regression RME consideration model may enhance the prediction accuracy and improve the model estimates, as well as our understanding to the impacts of various factors on RMEs’ consideration to provide O&M services to DWS in rural, low-resourced communities.

The regionalization of responsible management is no doubt promising to achieve the financial sustainability of RMEs. The question is what this consolidation looks like considering the local infrastructure settings and communities’ preferences, and what does it take to effectively operationalize this management model (e.g., creating legal structure, establishing affordable rates, developing a mechanism for bill collections). Future research is thus invited to further investigate these aspects, thereby supporting the water industry’s efforts toward moving to DWS approach that uses more cost-effective technologies and is more affordable to communities, especially rural, low-resourced communities. Additional research is also called for to explore the feasibility of considering other nonwater-wastewater service providers as potential RME types (e.g., electric utilities, electric cooperatives, solid waste management), as well as the exploration of energy sector literature (e.g., distributed renewable energy) to further inform this work. For instance, Murphy et al.^44^ suggests the use of rural electric cooperatives’ management model by any private or public entity interested in providing O&M services for DWS. As researchers continue to improve our understanding of various socio-technical aspects surrounding the responsible management of DWS, RMEs will be able to implement better-informed strategies for providing rural, low-resourced communities with effective, sustainable, and equitable wastewater services.

## Supporting Information Available

Major typologies of decentralized wastewater infrastructure systems. Existing management framework of decentralized wastewater systems. Typical activities performed by RME. Information about Black Belt counties. Survey questionnaire. Participated entities by states and respondents’ demographics. Distribution of survey responses by control variables. Correlation assessment. BL regression results for RME consideration model. Alternative BL regression RME consideration model. (PDF)

## Abbreviations

ADEM: Alabama Department of Environmental Management
ADPH: Alabama Department of Public Health
CDC: Community Development Corporation
CWSRF: Clean Water State Revolving Fund
DWS: Decentralized Wastewater Systems
OWTS: Onsite Wastewater Treatment Systems
O&M: Operations and Maintenance
PPP: Public-Private Partnership
RME: Responsible Management Entity
SDG: Sustainable Development Goal
US: United States
