# Supplementary material for: Sustainable Development in Rural Underserved Communities through Improved Responsible Management of Decentralized Wastewater Infrastructure: A Focus on the Alabama Black Belt
Source: Environ Sci Technol. 2024 Oct 10;58(42):18671–85. doi: 10.1021/acs.est.4c01170 (PMC11500419; doi:10.1021/acs.est.4c01170)
Supplement: Supplementary file 1 — es4c01170_si_001.pdf [file es4c01170_si_001.pdf]

Sustainable Development in Rural Underserved Communities through Improved Responsible Management of Decentralized Wastewater Infrastructure: A Focus on the Alabama Black Belt

Amal Bakchan, Ph.D.[*,1], *Kevin D. White*, Ph.D.[2]

[1] Assistant Professor, Dept. of Construction Science, Texas A&M University, 3137 TAMU, College Station, TX 77843, United States (corresponding author). ORCID: https://orcid.org/0000-0002-1709-5937. Email: bakchan@tamu.edu

[2] Emeritus Professor, Civil, Coastal and Environmental Engineering, University of South Alabama, 150 Student Services Drive, SHEC 3142, Mobile, AL 36688, United States; PH (251) 460-6174; email: kwhite@southalabama.edu

Number of Figures: 4

Number of Tables: 10

Table of Contents

1. MAJOR TYPOLOGIES OF DECENTRALIZED WASTEWATER INFRASTRUCTURE SYSTEMS S2

2. EXISTING MANAGEMENT FRAMEWORK OF DECENTRALIZED WASTEWATER SYSTEMS S3

3. TYPICAL ACTIVITIES PERFORMED BY RME S4

4. INFORMATION ABOUT BLACK BELT COUNTIES S5

5. SURVEY QUESTIONNAIRE S7

6. PARTICIPATED ENTITIES BY STATES AND RESPONDENTS' DEMOGRAPHICS..........S9

7. DISTRIBUTION OF SURVEY RESPONSES BY CONTROL VARIABLES S10

8. CORRELATION ASSESSMENT..........S11

9. BL REGRESSION RESULTS FOR RME CONSIDERATION MODEL S13

10. ALTERNATIVE BL REGRESSION RME CONSIDERATION MODEL S14

REFERENCES S16

1. Major Typologies of Decentralized Wastewater Infrastructure Systems

Wastewater infrastructure system typologies primarily vary from the conventional centralized systems to the decentralized approaches that include onsite wastewater treatment systems (OWTS) and clustered systems (see Figure S1). Centralized wastewater systems (Figure S1(a)) collect and treat large volume of wastewater for entire communities, where treated wastewater is often reused/disposed far from the generation point.[1,2] On the other hand, decentralized systems collect, treat, and reuse/dispose treated wastewater at or near the generation point.[1,3] More specifically, decentralized OWTS (Figure S1(b)) treat wastewater of individual homes or buildings,[2] whereas decentralized clustered systems (Figure S1(c)) involve collecting wastewater from multiple dwellings (e.g., 100+ homes) and conveying it to a shared treatment and dispersal system located somewhat near the dwellings.[2,4–6] Depending on the local context, decentralized clustered wastewater systems (with appropriate collection and treatment technologies) may be economically efficient compared to a typical centralized alternative (gravity collection and aerated treatment) and be cost-competitive with and more reliable than onsite septic systems.[7] For instance, clustered systems are especially favorable in areas that have poor soil conditions, high groundwater table, and/or adverse topography.[2]

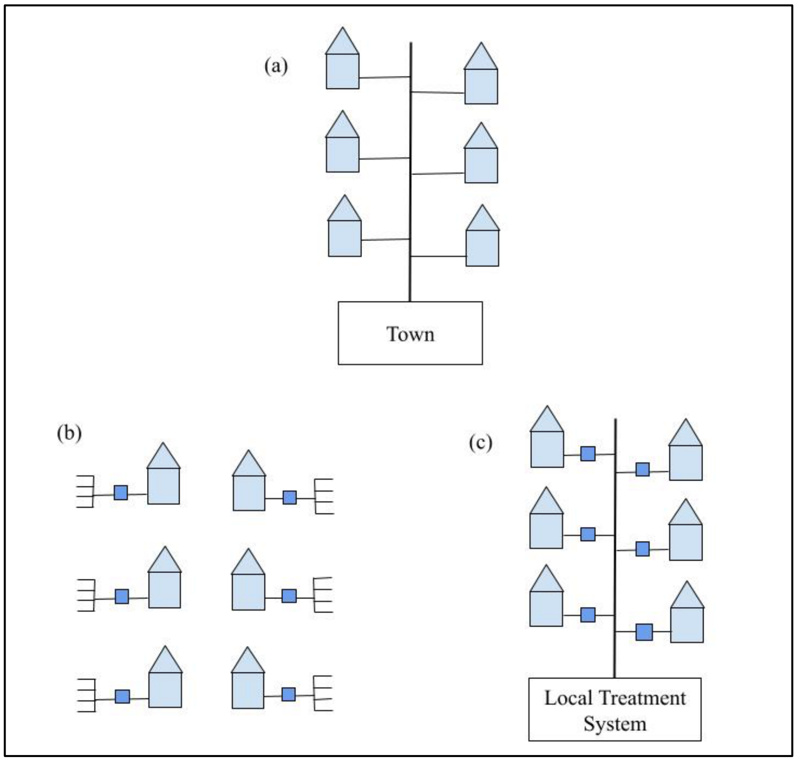

Figure S1. Illustration of major wastewater system typologies, denoting connection to existing sewer through (a) conventional centralized system. Households have local treatment through (b) permitted individual OWTS or (c) shared clustered system that collects wastewater from multiple dwellings. Small squares represent septic tanks that collect solids, with liquid joining a sewer main in typology (c) or continuing to a drainfield in typology (b).

It is important to note that multiple system typologies can be implemented within a single service area. For instance, in rural communities, there might be a combination of two or more of these approaches, where some houses may be connected to a centralized system, while others may have OWTS or be part of a clustered treatment system.[4] In such communities, even the centralized system typology (Figure S1(a)) would have a limited number of connections (e.g., 200-to-400 connections), despite the misconception that centralized systems are only used for serving large communities (e.g., 50-100 thousand people). Therefore, considering the definition of decentralized wastewater systems (DWS)—i.e., close proximity to the generation point—small municipal centralized systems in these rural communities might be classified as decentralized systems.

2. Existing Management Framework of Decentralized Wastewater Systems

According to US-EPA[8], decentralized management requirements vary based on the treatment system's complexity, as well as environmental sensitivity or public health concerns of an area. In this regard, a five-level conceptual decentralized systems management framework exists, ranging from approaches with least management controls—primarily adequate for conventional onsite septic systems that require little owner attention (homeowner awareness model)—to approaches with higher management restrictions;[8] see Figure S2 for further details.

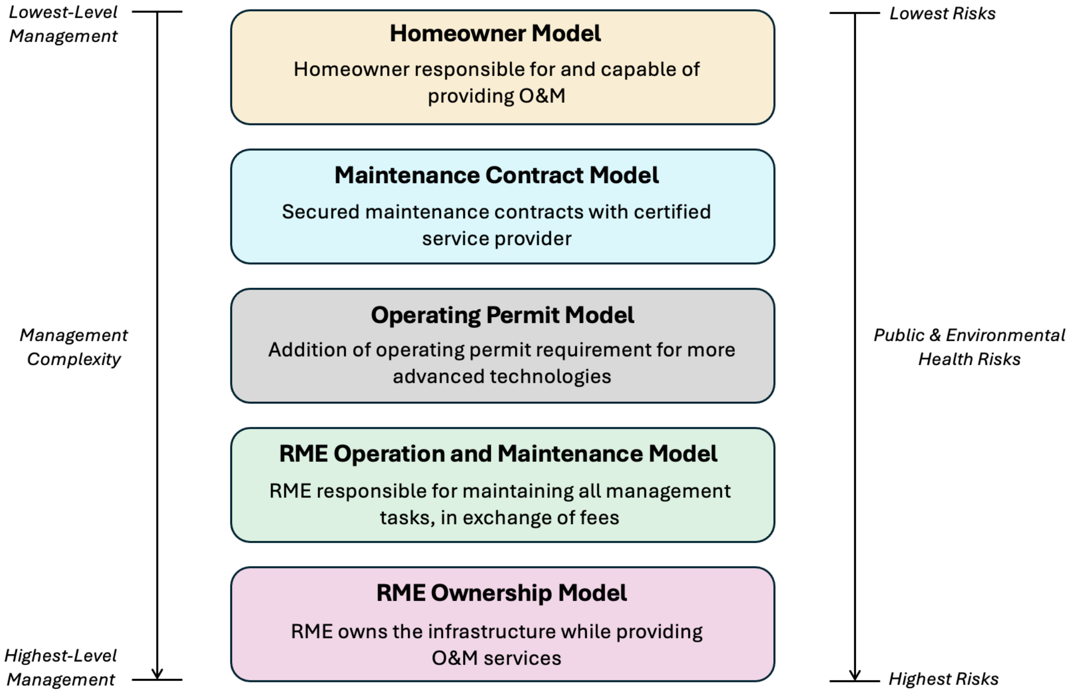

Figure S2. Five-level Management Framework of Decentralized Wastewater Infrastructures

3. Typical Activities Performed by RME

Table S1 summarizes the life-cycle series activities typically performed by RMEs when managing decentralized wastewater infrastructure systems.

Table S1. Typical activities performed by RMEs[6,8,9]

Activity	Description
System performance	• Establishing system performance and monitoring requirements to identify problems needing attention before failures occur
Operating permits	• Acquiring and maintain operating permits that comply with the local regulatory requirements related to the pumping, hauling/collection, treatment, and disposal<br>• E.g., The National Pollutant Discharge Elimination System (NPDES) permit is required for surface discharge of treated wastewater
Operator licensing	• Ensuring proper O&M and repairs to comply with performance criteria stipulated in the operating permit<br>• Obtaining appropriate operator certifications or licenses needed for achieving proper O&M practice,<br>• Arranging supplemental training for operators as needed
System compliance, tracking, and inventory	• Inspecting system compliance status<br>• Submitting compliance reports to the regulatory authority that proves the fulfillment of proper residuals management practices—from collection to disposal—as required by the operating permit
Billing and funding	• Defining the service charge fees that ensure RME's financial sustainability while being accepted by the community<br>• Handle the billing and collection of these fees<br>• Apply for funding whenever needed to support system operations
Public education and engagement	• Informing the homeowners of the appropriate and prohibited uses of the system<br>• Establishing community engagement when developing the responsible management services, especially charging rates, to ensure the public's acceptance and support to the RME

4. Information about Black Belt Counties

Table S2 shows the population and number of sewer connections in the 17 Black Belt counties.

Table S2. Population and Number of Connections in Black Belt Counties

County Name	County Population	% People w/ Sewer Service	Service Providers	Number of Connections	Design Flow Rate (MGD)
Barbour	24,686	68.91%	Town of Clayton Water and Sewer Board	1,127	0.4
			Town of Louisville	149	0.1
			City of Clio	545	0.4
			Eufaula Water Works	436	2.7
Bullock	10,101	52.46%	Town of Midway	181	0.15
			City of Union Springs Utility Board	1,745	2.25
Butler	19,448	23.14%	The Water Works & Sewer Board of the City of Georgiana	618	0.3
			Water Works and Sewer Board of the City of Greenville	1,018	2
Choctaw	12,589	22.62%	Utilities Board of the Town of Gilbertown	68	0.06
			Utilities Board of the Town of Butler	676	0.417
			North Choctaw Water & Sewer Authority	211	0.096
			Utilities Board of the Town of Pennington	80	0.095
Crenshaw	13,083	37.29%	Town of Rutledge	172	0.075
			The Water Works and Sewer Board of the City of Luverne	1,082	0.8
			Town of Brantley	345	0.2
			Town of Dozier	175	0.06
Dallas	37,196	68.83%	Dallas County Commission Delwood HCR Lagoon	73	0.06
			Dallas County Water and Sewer Authority	1,873	2
			Selma Water Works & Sewer Board	7,273	6
			City of Valley Grande	91	0.07
Greene	8,111	47.53%	Greene County Water and Sewer Authority	138	0.035
			City Of Eutaw	1,264	0.88
Hale	14,651	41.68%	City of Moundville	891	0.21
			Town Of Akron	129	0.08
			Utilities Board of the City of Greensboro	1,200	2

Lowndes	9,726	22.11%	Mosses Water, Sewer, and Fire Protection Authority	55	0.11
			Town Of Hayneville	255	0.18
			Fort Deposit Water Works & Sewer Board	473	0.24
Macon	18,068	23.66%	Utilities Board of the City of Tuskegee	1,273	3
			Town of Shorter	109	0.06
			Town of Notasulga	173	0.085
Marengo	18,863	53.67%	Utilities Board of the City of Linden	881	0.045
			The Water Works and Sewer Board of the City of Demopolis	2,800	2.65
Montgomery	226,361	89.76%	The Water Works & Sanitary Sewer Board of the City of Montgomery	72,800	60.59
			Pintlala Water Authority	15	0.01
			Clearwater Solutions	127	0.025
			Alabama Wastewater Systems, Inc.	945	2
Perry	8,923	15.41%	City Of Marion	500	0.5
			The Waterworks and Sewer Board of the City of Uniontown	Not listed	Not listed
Pike	33,009	67.77%	City of Troy	6,979	4.99
			City of Brundidge	1,091	0.6
Russell	57,961	62.69%	Water Works Board of the Town of Hurtsboro	122	0.26
			Phenix City Department of Public Utilities	13,091	6
Sumter	12,427	38.06%	Town Of Cuba	218	0.06
			City of York	455	0.6
			Sumter County Commission	189	0.06
			City of Livingston	600	0.8
			Industrial Development Authority of Sumter County	91	0.02
			Sumter County Sewer Authority	167	Not listed
Wilcox	10,373	54.56%	Town of Pine Hill	240	0.125
			City Of Camden	1,636	0.78
			Wilcox County Water Authority	182	0.03

5. Survey Questionnaire

Table S3 shows survey questions and answer options related to dependent, independent, and control variables used for binomial logistic (BL) regression modeling, as well as corresponding recoded categorical levels.

Table S3. (a) Questionnaire used for data collection, and (b) corresponding recoded categorical levels for BL regression modeling

Variable	(a) Survey Questionnaire		(b) BL Modeling
	Survey Question	Survey Answer	Categorical Level
**Dependent variable**			
RME consideration	Would you consider operating and maintaining multiple decentralized clustered wastewater systems that may span multiple jurisdictions and be several miles away?	• No<br>• Yes	• 0 – No<br>• 1 – Yes
**Independent variables: Socio-technical barriers**			
What are possible barriers that may prevent you from operating and managing small, alternative DWS? Please rate your concerns. If you think any of these listed reasons are not possible barriers, please select “Not a barrier”.		• Not a barrier<br>• Not concerning<br>• Somewhat concerning<br>• Neutral<br>• Concerning<br>• Very concerning	• 0 – Not concerning<br>• 1 – Concerning
Operator turnover	Difficulty to retain local skilled operators due to high turnover in rural areas, leading to limited expertise in the O&M of DWS		
Financial incentives	Limited financial incentives to manage new DWS		
Public funds	Difficulty to obtain public funds/capital for privately owned or managed systems		
Financial capacity	Limited communities’ financial capacity to pay for O&M		
Operational cost	Unclear operational cost of DWS		
Regulatory codes	Inflexible and prescriptive regulatory codes		
Liability concerns	Liability concerns with managing unfamiliar systems		
Organizational structures	Lack of necessary organizational structures for managing DWS		
Environmental awareness	Lack of communities’ awareness to risks associated with failing wastewater systems		
Equity concerns	Potential change in community socio-demographics and concerns of not meeting their actual needs		
**Control variables: RME structure and operational aspects**			

Entity type	Please specify the type of your entity.	• Public<br>• Private<br>• Non-profit<br>• Other	• 0 – Non-public<br>• 1 – Public
Management scale	Which scale do you think would be more feasible for the RME handling the DWS?	• Community<br>• County<br>• Regional<br>• State	• 1 – Community<br>• 2 – County<br>• 3 – Regional<br>• 4 – State
Service type	What type of services does your entity provide? Please specify all that apply.	• Water-only<br>• Wastewater-only<br>• Water-wastewater<br>• Hybrid [a]	• 0 – Wastewater not provided<br>• 1 – Wastewater provided
System size [b]	How many households do your systems serve?	• Less than 1,000<br>• 1,001-10,000<br>• 10,001-50,000<br>• More than 50,000	• 1 – Small<br>• 2 – Medium<br>• 3 – Large<br>• 4 – Very large
Location of operation [c]	In which state(s) does the entity operate? => mapped to whether it is the same as the location of proposed DWS (i.e., Alabama)	Open ended	• 0 – Different<br>• 1 – Same
Decentralized service operation	Do you currently provide O&M services to DWS?	• No<br>• Yes	• 0 – No<br>• 1 – Yes
Centralized service operation	Do you currently provide O&M services to centralized wastewater systems?	• No<br>• Yes	• 0 – No<br>• 1 – Yes
Operational flexibility [d]	Can you manage DWS that may be located outside the typical service area?	• No<br>• Yes	• 0 – No<br>• 1 – Yes
	Would you be willing to accept effluent sewer (no solid; liquid only) from another nearby community, if system capacity allows?	• No<br>• Yes	

[a] Hybrid includes water-sector services (e.g., water, wastewater) along with at least one other service (e.g., gas, electricity, solid waste).

[b] Number of households in each system size category[10]: Small (less than 1,000), medium (1,001-to-10,000), large (10,001-to-50,000), very large (more than 50,000).

[c] Mapped to whether it is the same as the location of the proposed DWS (i.e., Alabama).

[d] Answer of this variable is based on two survey questions; it is mapped to “1 – Yes” if the answer to any of these two questions is “Yes”.

6. Participated Entities by States and Respondents' Demographics

Table S4 shows the number of participated entities across the 27 states, totaling 121 responses. The majority of responses (36%) are from entities operating in Alabama, followed by Texas (10%).

Table S4. Number of participated entities across the 27 states

State	Number of Entities
Alabama	43
Arkansas	4
California	2
Colorado	1
Connecticut	4
Florida	2
Idaho	3
Iowa	2
Kansas	2
Kentucky	3
Maryland	1
Massachusetts	2
Missouri	4
Montana	1
Nebraska	2
Nevada	3
New Jersey	3
New York	2
Pennsylvania	3
Rhode Island	2
Tennessee	4
Texas	12
Vermont	2
Virginia	4
Washington	3
Wisconsin	3
Wyoming	4
Total	*121*

Figure S3 shows survey respondents' demographics related to gender, race/ethnicity, and years of professional experience. For instance, 59% of respondents have more than 20 years of experience.

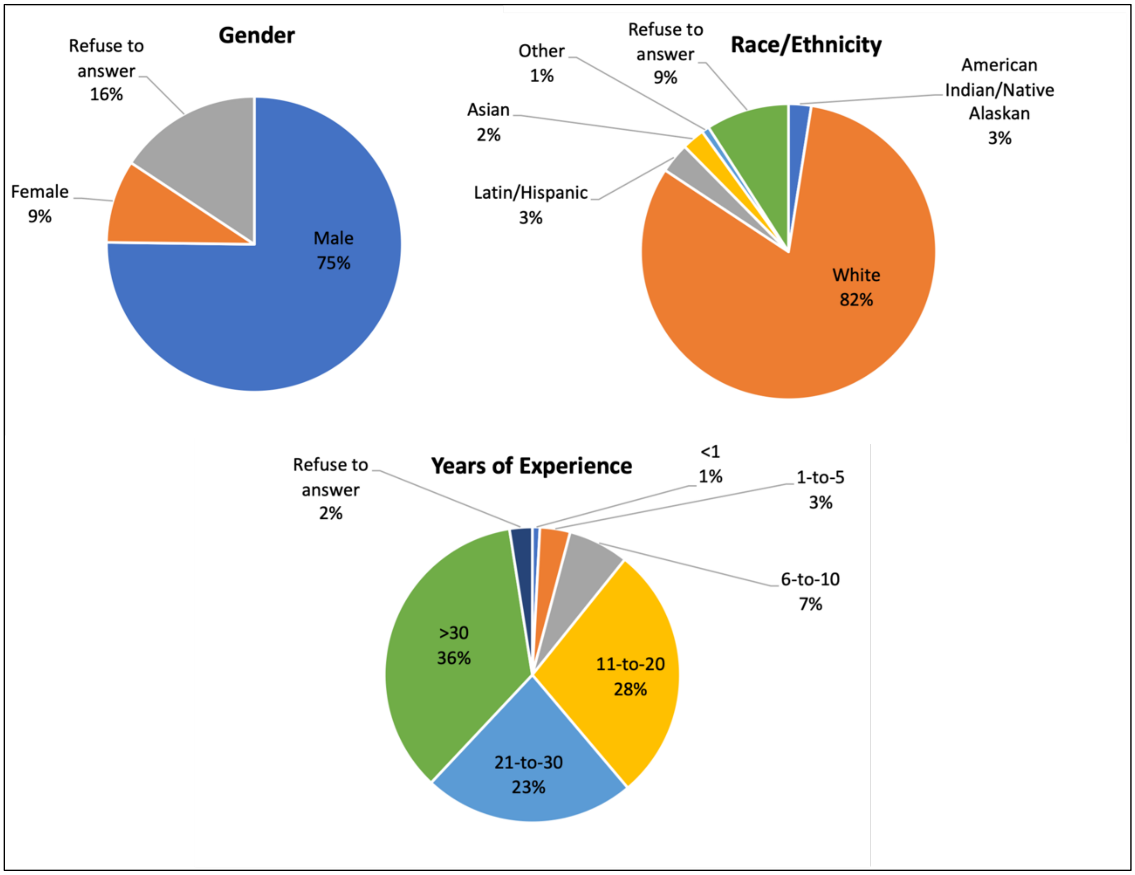

Figure S3. Survey Respondents' Demographics, by gender, race/ethnicity, and years of experience.

7. Distribution of Survey Responses by Control Variables

Figure S4 shows the distribution of survey responses by the seven control variables, i.e., entity type, management scale, service type, system size, location of operation, decentralized service operation, centralized service operation, and operational flexibility. For instance, over 70% of entities that participated in the survey provides wastewater services as part of their operations, with 27% only serving wastewater systems. Additionally, 77% of the entities have operational flexibility that allows them to operate outside their service area and/or accept effluent from nearby communities.

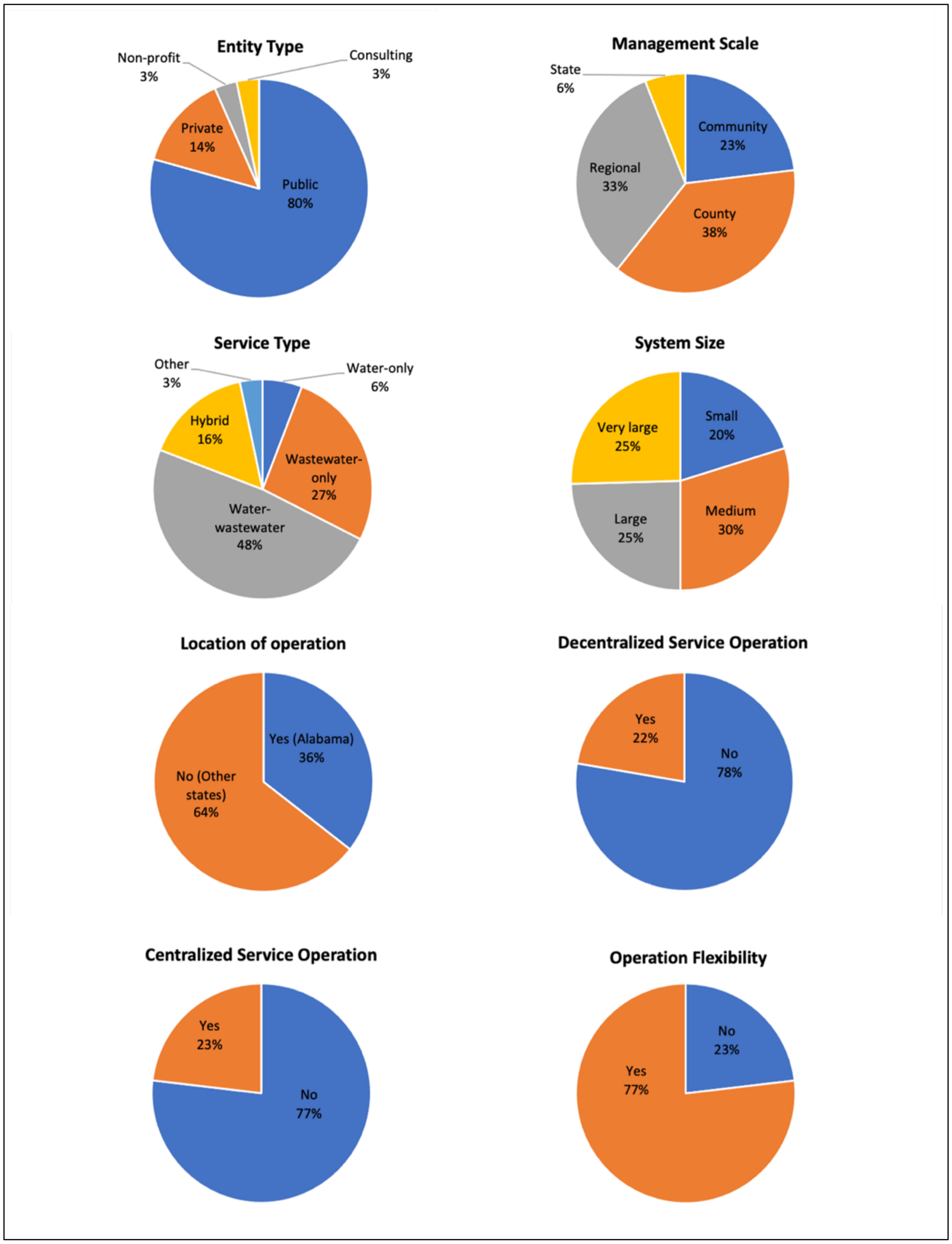

Figure S4. Distribution of survey responses by the eight control variables.

8. Correlation Assessment

Table S5 shows the correlation matrix, where several independent and control variables exhibited collinearity issues (correlations > 0.5).[11] After excluding "financial incentives", "organizational structures", "service type", and "centralized service operation", the final generated RME consideration model (Table S6) indicated no collinearity concerns (VIF < 5)[12].

Table S5. Correlations in predictors of RME consideration model

Variable	*OT*	*FI*	*PF*	*FC*	*OC*	*RC*	*LC*	*OS*	*EA*	*EC*	*ET*	*MS*	*ST*	*SS*	*LO*	*DSO*	*CSO*	*OF*
**Independent variables: Socio-technical barriers**																		
Operator turnover	–																	
Financial incentives	0.53	–																
Public funds	0.31	0.57	–															
Financial capacity	0.26	0.47	0.44	–														
Operational cost	0.10	0.22	0.25	0.31	–													
Regulatory codes	0.27	0.18	0.16	0.30	0.39	–												
Liability concerns	0.24	0.27	0.32	0.26	0.47	0.41	–											
Organizational structures	0.34	0.27	0.19	0.27	0.41	0.55	0.53	–										
Environmental awareness	0.21	0.24	0.18	0.37	0.18	0.33	0.35	0.33	–									
Equity concerns	0.12	0.08	0.08	0.15	0.06	0.03	0.25	0.11	0.31	–								
**Control variables: RME structure and operational aspects**																		
Entity type	0.0003	-0.08	0.004	-0.21	0.26	0.15	0.16	0.04	-0.11	0.04	–							
Management scale	-0.006	0.09	-0.03	0.16	-0.02	-0.05	-0.05	-0.08	-0.03	-0.16	-0.44	–						
Service type	-0.17	-0.07	-0.04	0.15	0.18	0.08	0.21	0.12	0.24	0.21	-0.01	0.19	–					
System size	-0.14	-0.02	-0.07	-0.12	0.02	0.02	-0.01	-0.07	-0.12	0.07	0.39	-0.002	0.16	–				
Location of operation	0.21	0.13	0.13	0.05	0.14	0.04	0.10	0.23	0.03	0.05	-0.19	-0.05	-0.03	-0.23	–			
Decentralized service operation	-0.12	0.04	-0.07	0.06	-0.27	-0.12	-0.05	-0.10	0.12	0.08	-0.27	0.16	0.17	0.05	0.04	–		
Centralized service operation	-0.22	-0.17	-0.04	0.05	0.26	0.15	0.16	0.04	0.15	0.04	0.39	-0.002	0.59	0.34	-0.14	-0.02	–	
Operational flexibility	-0.08	0.08	0.14	0.19	0.17	0.17	0.08	-0.11	0.14	-0.01	0.04	0.05	0.34	-0.01	-0.20	0.03	0.42	–

Note: *OT* = Operator turnover, *FI* = Financial incentives, *PF* = Public funds, *FC* = Financial capacity, *OC* = Operational cost, *RC* = Regulatory codes, *LC* = Liability concerns, *OS* = Organizational structures, *EA* = Environmental awareness, *EC* = Equity concerns, *ET* = Entity type, *MS* = Management scale, *ST* = Service type, *SS* = System size, *LO* = Location of operation, *DSO* = Decentralized service operation, *CSO* = Centralized service operation, *OF* = Operational flexibility.

Table S6. VIF values for independent and control variables of final RME consideration model

Variable	VIF	VIF 95% CI	Increased SE	Tolerance	Tolerance 95% CI
**Independent variables: Socio-technical barriers**					
Operator turnover	1.88	[1.55, 2.41]	1.37	0.53	[0.42, 0.64]
Financial capacity	1.60	[1.35, 2.04]	1.27	0.62	[0.49, 0.74]
Public funds	1.60	[1.35, 2.04]	1.26	0.63	[0.49, 0.74]
Operational cost	2.06	[1.68, 2.64]	1.43	0.49	[0.38, 0.59]
Regulatory codes	1.90	[1.57, 2.44]	1.38	0.53	[0.41, 0.64]
Liability concerns	2.03	[1.66, 2.60]	1.42	0.49	[0.38, 0.60]
Environmental awareness	1.47	[1.26, 1.87]	1.21	0.68	[0.53, 0.80]
Equity concerns	1.51	[1.28, 1.92]	1.23	0.66	[0.52, 0.78]
**Control variables: RME structure and operational aspects**					
Entity type	2.10	[1.71, 2.69]	1.45	0.48	[0.37, 0.59]
Management scale	2.63	[2.10, 3.41]	1.62	0.38	[0.29, 0.48]
System size	2.54	[2.04, 3.29]	1.59	0.39	[0.30, 0.49]
Location of operation	1.34	[1.17, 1.72]	1.16	0.74	[0.58, 0.86]
Decentralized service operation	1.37	[1.19, 1.75]	1.17	0.73	[0.57, 0.84]
Operational flexibility	1.14	[1.04, 1.55]	1.07	0.88	[0.65, 0.96]

9. BL Regression Results for RME Consideration Model

Table S7 summarizes the BL regression analysis results.

Table S7. BL regression results for RME consideration model [a]

Variable	β_i	Std. Error	z	p
intercept	-2.42	1.73	-1.40	0.16
**Independent variables: Socio-technical barriers**				
Operator turnover	-2.01	0.71	-2.84	0.005^{***}
Financial capacity	0.94	0.91	1.03	0.30
Public funds	-0.26	0.68	-0.39	0.69
Operational cost	-0.70	0.73	-0.97	0.33
Regulatory codes	1.53	0.70	2.19	0.03^{**}
Liability concerns	0.46	0.73	0.64	0.52
Environmental awareness	-0.44	0.64	-0.68	0.49
Equity concerns	1.06	0.65	1.64	0.101
**Control variables: RME structure and operational aspects**				
Entity type	-2.05	0.97	-2.11	0.03^{**}
Management scale – county	1.82	0.82	2.23	0.02^{**}
Management scale – regional	0.86	0.89	0.97	0.33
Management scale – state	1.45	1.34	1.08	0.28

System size – medium	-0.39	0.85	-0.46	0.64
System size – large	-0.29	0.91	-0.31	0.75
System size – very large	-1.05	0.97	-1.09	0.28
Location of operation	0.66	0.62	1.07	0.28
Decentralized service operation	1.52	0.70	2.16	0.03**
Operational flexibility	2.44	1.21	2.01	0.04**

[a] BL regression analysis. *$p < 0.1$. **$p < 0.05$. ***$p < 0.01$.

Model information: Null deviance = 150.05 on 113 degrees of freedom; Residual deviance= 96.37 on 95 degrees of freedom; AIC = 134.37; Number of Fisher scoring iterations = 5; McFadden's *pseudo*-R^2 = 0.36.

10. Alternative BL Regression RME Consideration Model

The "public funds" variable is excluded from the RME consideration model—referred here to as *alternative* RME consideration model.

All VIF values for independent and control variables of the alternative RME consideration model are less than 5, indicating no collinearity issues (see Table S8). Table S9 shows the BL Regression results for the alternative RME consideration model when excluding the "public funds" independent variable. The odds ratios for this model are shown in Table S10.

Table S8. VIF values for independent and control variables of alternative RME consideration model

Variable	VIF	VIF 95% CI	Increased SE	Tolerance	Tolerance 95% CI
**Independent variables: Socio-technical barriers**					
Operator turnover	1.88	[1.55, 2.41]	1.37	0.53	[0.42, 0.64]
Financial capacity	1.60	[1.35, 2.04]	1.27	0.62	[0.49, 0.74]
Public funds	1.60	[1.35, 2.04]	1.26	0.63	[0.49, 0.74]
Operational cost	2.06	[1.68, 2.64]	1.43	0.49	[0.38, 0.59]
Regulatory codes	1.90	[1.57, 2.44]	1.38	0.53	[0.41, 0.64]
Liability concerns	2.03	[1.66, 2.60]	1.42	0.49	[0.38, 0.60]
Environmental awareness	1.47	[1.26, 1.87]	1.21	0.68	[0.53, 0.80]
Equity concerns	1.51	[1.28, 1.92]	1.23	0.66	[0.52, 0.78]
**Control variables: RME structure and operational aspects**					
Entity type	2.10	[1.71, 2.69]	1.45	0.48	[0.37, 0.59]
Management scale	2.63	[2.10, 3.41]	1.62	0.38	[0.29, 0.48]
System size	2.54	[2.04, 3.29]	1.59	0.39	[0.30, 0.49]
Location of operation	1.34	[1.17, 1.72]	1.16	0.74	[0.58, 0.86]
Decentralized service operation	1.37	[1.19, 1.75]	1.17	0.73	[0.57, 0.84]
Operational flexibility	1.14	[1.04, 1.55]	1.07	0.88	[0.65, 0.96]

Table S9. BL regression results for the alternative RME consideration model [a]

Variable	β_i	Std. Error	z	p
intercept	-2.39	1.71	-1.39	0.16
**Independent variables: Socio-technical barriers**				
Operator turnover	-2.06	0.70	-2.96	0.003***
Financial capacity	0.81	0.84	0.96	0.34
Operational cost	-0.67	0.72	-0.93	0.35
Regulatory codes	1.57	0.69	2.26	0.02**
Liability concerns	0.39	0.70	0.56	0.58
Environmental awareness	-0.44	0.64	-0.69	0.49
Equity concerns	1.07	0.65	1.66	0.09*
**Control variables: RME structure and operational aspects**				
Entity type	-2.06	0.97	-2.12	0.03**
Management scale - county	1.81	0.82	2.22	0.03**
Management scale - regional	0.89	0.88	1.01	0.31
Management scale - state	1.41	1.33	1.06	0.29
System size – medium	-0.40	0.85	-0.47	0.64
System size – large	-0.28	0.91	-0.31	0.76
System size – very large	-1.11	0.96	-1.15	0.25
Location of operation	0.62	0.61	1.01	0.31
Decentralized service operation	1.54	0.70	1.01	0.03**
Operational flexibility	2.39	1.20	1.99	0.04**

[a] BL regression analysis. $^{*}p < 0.1$. $^{**}p < 0.05$. $^{***}p < 0.01$.

Model information: Null deviance = 150.05 on 113 degrees of freedom; Residual deviance= 96.52 on 96 degrees of freedom; AIC = 132.52; Number of Fisher scoring iterations = 5; McFadden's *pseudo*-R^2 = 0.36.

Table S10. Odds ratio results for the alternative RME consideration model [a]

Variable	Odds Ratio	2.5%	97.5%	*p*
intercept	0.09	0.002	2.01	0.16
**Independent variables: Socio-technical barriers**				
Operator turnover	0.13	0.03	0.46	0.003***
Financial capacity	2.24	0.47	13.21	0.34
Operational cost	0.51	0.12	2.09	0.35
Regulatory codes	4.80	1.30	20.55	0.02**
Liability concerns	1.48	0.37	5.99	0.58
Environmental awareness	0.64	0.18	2.23	0.49
Equity concerns	2.23	0.85	11.18	0.09*
**Control variables: RME structure and operational aspects**				
Entity type	0.13	0.02	0.77	0.03**
Management scale - county	6.14	1.36	34.79	0.03**
Management scale - regional	2.43	0.43	14.59	0.31
Management scale - state	4.08	0.28	58.63	0.29
System size – medium	0.67	0.13	3.90	0.64
System size – large	0.75	0.13	4.83	0.76
System size – very large	0.33	0.05	2.26	0.25
Location of operation	1.85	0.56	6.29	0.31
Decentralized service operation	4.68	1.23	19.78	0.03**
Operational flexibility	10.94	1.50	249.54	0.04**

[a] BL regression analysis – odds ratios at 95% CI. $^{*}p < 0.1$. $^{**}p < 0.05$. $^{***}p < 0.01$.

References

(1) US-EPA. *Primer for Municipal Wastewater Treatment Systems (EPA 832-R-04-001)*; 2004.

(2) Massoud, M. A.; Tarhini, A.; Nasr, J. A. Decentralized Approaches to Wastewater Treatment and Management: Applicability in Developing Countries. *J. Environ. Manage.* **2009**, *90* (1), 652–659. https://doi.org/10.1016/j.jenvman.2008.07.001.

(3) Tchobanogious, G.; Ruppe, L.; Leverenz, H.; Darby, J. Decentralized Wastewater Management: Challenges and Opportunities for the Twenty-First Century. *Water Sci. Technol. Water Supply* **2004**, *4* (1), 95–102. https://doi.org/10.2166/ws.2004.0011.

(4) Buchanan, J. *Decentralized Systems: Performance and Cost of Decentralized Unit Processes*; 2010. http://www.werf.org/c/decentralizedcost/t4_constructed_wetla.aspx.

(5) Sheehan, K. A. Decentralized Wastewater Treatment in Georgia : Benefits and Management Needs. In *Georgia Water Resources Conference*; University of Georgia: Athens, Georgia, 2011.

(6) US-EPA. *Handbook for Managing Onsite and Clustered (Decentralized) Wastewater Treatment Systems: An Introduction to Management Tools and Information for Implementing EPA's Management Guidelines (EPA No. 832-B-05-001)*; 2005. https://nepis.epa.gov/Exe/ZyNET.exe/20017K2G.TXT?ZyActionD=ZyDocument&Client=EPA&Index=2000+Thru+2005&Docs=&Query=&Time=&EndTime=&SearchMethod=1&TocRestrict=n&Toc=&TocEntry=&QField=&QFieldYear=&QFieldMonth=&QFieldDay=&IntQFieldOp=0&ExtQFieldOp=0&XmlQuery=.

(7) Schwetschenau, S. E.; Kovankaya, Y.; Elliott, M. A.; Allaire, M.; White, K. D.; Lall, U. Optimizing Scale for Decentralized Wastewater Treatment: A Tool to Address Failing Wastewater Infrastructure in the United States. *ACS ES T Eng.* **2023**, *3* (1), 1–14. https://doi.org/10.1021/acsestengg.2c00188.

(8) US-EPA. *Voluntary National Guidelines for Management of Onsite and Clustered (Decentralized) Wastewater Treatment Systems (EPA 832-B-03-001)*; 2003.

(9) US-EPA. *Using a Responsible Management Entity (RME) to Manage Tribal Onsite (Septic) Wastewater Treatment Systems (EPA No. 830K17003)*; 2018. https://www.epa.gov/sites/default/files/2018-02/documents/tribal_rme_guidance_508c.pdf.

(10) US-EPA. *2006 Community Water System Survey - Volume I: Overview (EPA-815-R-09-001)*; 2006.

(11) Chambers, J. M. *Statistical Models in S*; Chambers, J. M., Hastie, T. J., Eds.; Wadsworth & Brooks/Cole, 1992. https://doi.org/https://doi-org.ezproxy.lib.utexas.edu/10.1201/9780203738535.

(12) James, G.; Witten, D.; Hastie, T.; Tibshirani, R. *An Introduction to Statistical Learning: With Applications in R.*; Springer: New York, 2013.
